# Fecal microbiota transplantation can improve cognition in patients with cognitive decline and *Clostridioides difficile* infection

**DOI:** 10.18632/aging.204230

**Published:** 2022-08-16

**Authors:** Soo-Hyun Park, Jung-Hwan Lee, Jun-Seob Kim, Tae Jung Kim, Jongbeom Shin, Jae Hyoung Im, Boram Cha, Suhjoon Lee, Kye Sook Kwon, Yong Woon Shin, Sang-Bae Ko, Seong Hye Choi

**Affiliations:** 1 Department of Neurology, Department of Critical Care Medicine, Department of Hospital Medicine, Inha University Hospital, Incheon 22332, Republic of Korea; 2 Division of Gastroenterology, Department of Internal Medicine, Department of Hospital Medicine, Inha University Hospital, Incheon 22332, Republic of Korea; 3 Department of Nano-Bioengineering, Incheon National University, Incheon 22012, Republic of Korea; 4 Department of Neurology and Department of Critical Care Medicine, Seoul National University Hospital, Seoul 03080, Republic of Korea; 5 Division of Gastroenterology, Department of Internal Medicine, Inha University School of Medicine, Incheon 22332, Republic of Korea; 6 Division of Infectious Diseases, Department of Internal Medicine, Inha University School of Medicine, Incheon 22332, Republic of Korea; 7 Department of Neurology, Inha University School of Medicine, Incheon 22332, Republic of Korea

**Keywords:** fecal microbiota transplantation, gut microbiome, brain-gut-axis, cognitive function, Alzheimer’s dementia

## Abstract

After fecal microbiota transplantation (FMT) to treat *Clostridioides difficile* infection (CDI), cognitive improvement is noticeable, suggesting an essential association between the gut microbiome and neural function. Although the gut microbiome has been associated with cognitive function, it remains to be elucidated whether fecal microbiota transplantation can improve cognition in patients with cognitive decline.

The study included 10 patients (age range, 63–90 years; female, 80%) with dementia and severe CDI who were receiving FMT. Also, 10 patients (age range, 62–91; female, 80%) with dementia and severe CDI who were not receiving FMT. They were evaluated using cognitive function tests (Mini-Mental State Examination [MMSE] and Clinical Dementia Rating scale Sum of Boxes [CDR-SB]) at 1 month before and after FMT or antibiotics treatment (control group). The patients’ fecal samples were analyzed to compare the composition of their gut microbiota before and 3 weeks after FMT or antibiotics treatment.

Ten patients receiving FMT showed significantly improvements in clinical symptoms and cognitive functions compared to control group. The MMSE and CDR-SB of FMT group were improved compare to antibiotics treatment (MMSE: 16.00, median, 13.00–18.00 [IQR] vs. 10.0, median, 9.8–15.3 [IQR]); CDR-SB: 5.50, median, 4.00–8.00 [IQR]) vs. 8.0, median, 7.9–12.5, [IQR]). FMT led to changes in the recipient’s gut microbiota composition, with enrichment of *Proteobacteria* and *Bacteroidetes*. Alanine, aspartate, and glutamate metabolism pathways were also significantly different after FMT.

This study revealed important interactions between the gut microbiome and cognitive function. Moreover, it suggested that FMT may effectively delay cognitive decline in patients with dementia.

## INTRODUCTION

The importance of the bidirectional communication between the gut and central nervous system (the brain-gut-microbiome axis) is continually growing with our greater understanding of the process. Microbiota is involved in the pathophysiology of various cellular mechanisms, such as immune system, homeostasis, and disease, and is implicated in both health and disease [1, 2]. Particularly, healthy individuals present distinct human gut microbiota compositions compared to those with several neurological diseases, including Parkinson’s disease, Alzheimer’s disease (AD), autism spectrum disorder, multiple sclerosis, and amyotrophic lateral sclerosis [2, 3]. Changes in the diversity and richness of gut microbiota are correlated with disease progression, particularly with diminished cognitive function [4]. Furthermore, there is an interplay between gastrointestinal pathology and neuropsychiatric conditions [5]. In other words, if alteration of microbial species may induce immune signaling, it results in the change in host homeostasis and CNS disease progression. Therefore, the clinical features and development of various neurological disorders may be influenced by gut microbiota. Thus, fecal microbiota transplantation (FMT), in which fecal solution from a healthy donor is administered into a patient’s intestinal tract, may provide therapeutic benefit to patients.

Previous animal studies have demonstrated that FMT can improve emotional behaviors, learning, memory, and recognition [6]. Cognitive decline may occur in transplanted adult mice after FMT for spatial learning and memory through changes in the hippocampal synaptic plasticity-associated and neurotransmission-related proteins [7, 8]. These microbially derived changes may intrinsically regulate the host homeostasis, including the blood-brain barrier and intestinal integrity after FMT [6, 9]. However, no association between FMT and cognitive improvement has been established in humans. This study aims to establish that FMT leads to significant improvements in cognitive performance and discuss the relationship between cognition and the gut microbiome.

## RESULTS

### Clinical overview and cognitive function differences before and after FMT

Among 10 patients, 5 representative cases are briefly described below for easy understanding of this study.

**Figure 1:**
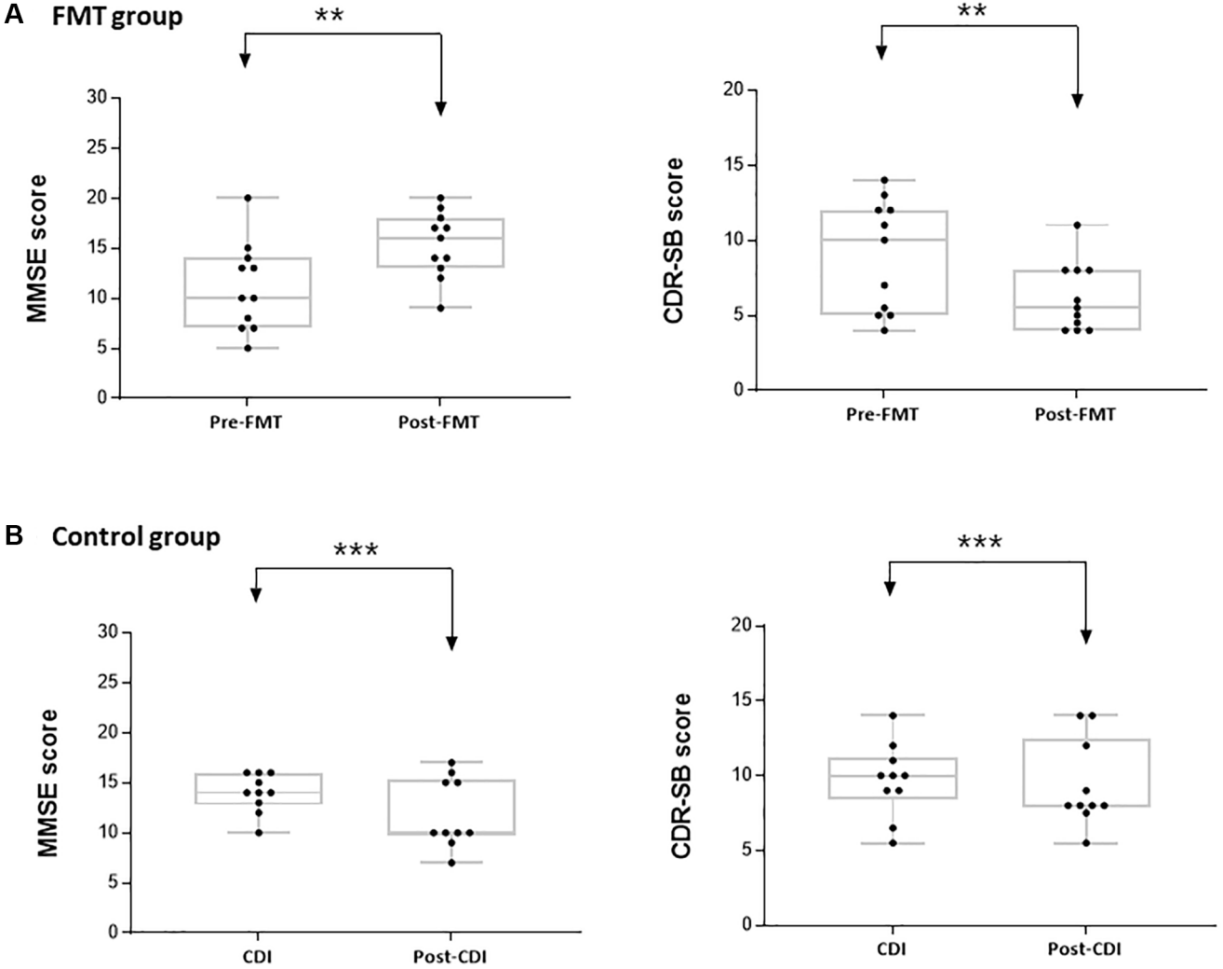
**Mini-mental state examination (MMSE) and clinical dementia rating scale sum of boxes (CDR-SB) scores in the study groups.** (**A**) Difference in the MMSE and CDR-SB scores before and after FMT. (**B**) Difference in the MMSE and CDR-SB scores before and after CDI. ^**^P < 0.05, ^***^P > 0.05.

Case 1: An 85-year-old woman was admitted to the intensive care unit for intracranial aneurysmal coiling treatment (Table 1). Subsequently, she suffered from diarrhea and was diagnosed with CDI. Antibiotic treatment, including vancomycin and metronidazole, failed. She had been experiencing a gradual decline in memory and cognition and was taking donepezil (10 mg) for AD. The patient scored 8 and 10 on her recent Mini-Mental State Examination (MMSE) and Clinical Dementia Rating Scale Sum of Boxes (CDR-SB) scores, respectively, while the Glasgow Coma Scale (GCS) score was 14. She no longer appeared to enjoy socializing and required considerable assistance with basic tasks, such as food preparation, bathing, and taking medication. She underwent FMT after 1 month, and her MMSE and CDR-SB scores improved to 13 and 8, respectively, and the GCS score was 14 (Table 2, Figure 1, and Supplementary Table 1). Following FMT, her severe gastrointestinal symptoms improved, and a stool test for CDI was negative.

**Table 1:** Clinical characteristics of the study participants.

**Characteristics**	**Value**		
	**FMT group (*N* = 10)**	**Control group (*N* = 10)**	***P* value**
**Sex, female, *n* (%)**	8 (80)	8 (80)	0.71
**Age, years, median (IQR)**	76 (63–90)	77 (62–91)	
**mRS, median (IQR)**	3 (1–4)	3 (1–4)	1.00
**Geriatric Depression Scale, median (IQR)**	17 (13–22)	16 (10–23)	0.84
**Nutrition supplement status**			0.22
Oral feeding, *n* (%)	9 (90)	8 (80)	
Enteral feeding, *n* (%)	1 (10)	2 (20)	
Peripheral feeding, *n* (%)	0 (0)	0 (0)	
**Presenting symptoms of CDI**			0.79
Diarrhea, *n* (%)	9 (90)	9 (90)	
Abdominal pain, *n* (%)	4 (40)	5 (50)	
Fever, *n* (%)	4 (40)	4 (40)	
**Antibiotics for CDI**			0.80
Vancomycin, *n* (%)	2 (20)	3 (30)	
Metronidazole, *n* (%)	2 (20)	2 (20)	
Vancomycin + metronidazole, *n* (%)	6 (60)	5 (50)	
FMT dose, g	60	–	–
**Complications after FMT**		–	–
None, *n* (%)	8 (80)		
Nausea, *n* (%)	1 (10)		
Abdominal pain, *n* (%)	1 (10)		
Fever, *n* (%)	1 (10)		
**Medication for dementia**			0.50
Donepezil, *n* (%)	7 (77.8)	6 (60)	
Memantine, *n* (%)	1 (11.1)	2 (20)	
Donepezil + Memantine, *n* (%)	1 (11.1)	1 (10)	
**Duration of dementia, years, median (IQR)**	3.5 (1.8–5.3)	3.8 (1.7–5.4)	0.73

Abbreviations: mRS: modified Rankin Scale; FMT: fecal microbiota transplantation; CDI: *Clostridioides difficile* infection; IOR: interquartile range.

**Table 2:** Cognitive function difference between the FMT and control groups.

**Cognitive function test**	**FMT group**				
	**Before FMT, median (IQR)**	**After FMT, median (IQR)**	**Before-After, mean (SD)**	***P* value^a^**	***P* value^b^**
**MMSE**	10.0 (7.0–14.0)	16.0 (13.0–18.0)	4.7 (1.6)	0.010	0.005
**CDR-SB score**	10.0 (5.0–12.0)	5.5 (4.0–8.0)	3.1 (2.6)	0.048	0.005
**Cognitive function test**	**Control group**				
	**Before antibiotics treatment, median (IQR)**	**After antibiotics treatment, median (IQR)**	**Before-After. mean (SD)**	***P* value^a^**	***P* value^b^**
**MMSE**	14.0 (12.8–16.0)	10.0 (9.8–15.3)	2.1 (2.8)	0.206	0.072
**CDR-SB score**	10.0 (8.4–11.3)	8.0 (7.9–12.5)	0.3 (1.3)	0.492	0.470

Abbreviations: FMT: fecal microbiota transplantation; MMSE: Mini-mental state examination; CDR-SB score: Clinical Dementia Rating Scale Sum of Boxes Score; IQR: interquartile range; CDI: *Clostridioides difficile* infection; SD: standard deviation. ^a^Kruskal-Wallis H. ^b^Wilcoxon signed rank.

Case 2: A 73-year-old woman with a history of AD, acute kidney injury, and atrial fibrillation was admitted for diarrhea and fever (Table 1). She was diagnosed with CDI, and intravenous vancomycin treatment failed. She also presented with AD, including depression, memory loss, and flattened affect. She took memantine (20 mg) and had MMSE and CDR-SB scores of 13 and 7, respectively, and GCS score of 13. After undergoing FMT, her MMSE and CDR-SB scores improved to 17 and 6, respectively, and the GCS score was 14 (Table 2, Figure 1, and Supplementary Table 1). Her severe gastrointestinal discomfort improved with negative CDI stool results 1 week after FMT.

Case 3: A 90-year-old woman with a history of AD, diabetes mellitus, hypertension, and chronic kidney disease was admitted for diarrhea and fever (Table 1). On hospital day 3, she was diagnosed with CDI. She was treated unsuccessfully with vancomycin with metronidazole. She was taking donepezil (10 mg) for AD. Her most recent MMSE and CDR-SB scores were 15 and 5.5, respectively, and GCS score was 14. After FMT, her MMSE score improved to 18 and CDR-SB score to 5 (Table 2, Figure 1). She no more experienced diarrhea and fever, with a negative CDI stool test after FMT. Three months after the first FMT, she suffered from diarrhea, fever (38.0°C), continuous abdominal pain, and progressively worsening conditions. She was re-diagnosed with a recurrent severe CDI with a positive CDI stool test, and was unsuccessfully treated with antibiotics. She underwent a second FMT with the same modalities as the first FMT. Her MMSE and CDR-SB scores before the second FMT procedure were 20 and 5.5, respectively, which improved immediately after the first FMT. One week after the second FMT, her severe gastrointestinal discomfort improved with negative CDI stool results. Nevertheless, her scores were stable (MMSE 20, CDR-SB 4). However, no difference was observed in GCS scores before and after FMT (Supplementary Table 1).

Case 4: A 63-year-old man with a history of diabetes mellitus, hypertension, stroke, and vascular dementia was admitted for pneumonia with dyspnea (Table 1). On hospital day 62, he was diagnosed with CDI and treated with oral metronidazole. Because of additional unsuccessful treatment with vancomycin, he underwent FMT. Before FMT, he presented with depression and memory loss, and his MMSE and CDR-SB scores were 14 and 5, respectively, and GCS score was 14. After FMT, his MMSE and CDR-SB scores improved to 19 and 4, respectively (Table 2, Figure 1). However, GCS was not different compared to that before FMT (Supplementary Table 1). He reported a marked improvement from previous symptoms.

Case 5: An 84-year-old woman with a history of ischemic cardiomyopathy and AD was admitted for type 2 acute respiratory failure (Table 1). On hospital day 7, CDI was diagnosed and treated with vancomycin and metronidazole. Because of additional unsuccessful treatment with oral antibiotics, she underwent FMT. Before FMT, she presented with depression and memory loss, and her MMSE and CDR-SB scores were 5 and 11, respectively. After FMT, her MMSE and CDR-SB scores improved to 12 and 8, respectively (Table 2, Figure 1). Following FMT, her stool test for CDI was negative.

### Baseline characteristics

Baseline characteristics of the study population are provided in Table 1. Subjects with dementia and severe CDI suffered from CDI symptoms, such as abdomen pain, diarrhea, vomiting, and fever. They were divided into two groups: those with FMT (FMT group) and without FMT (control group). In the FMT group, there were 8 women and 2 men; the median age at the time of FMT was 76 years (range, 63–90 years). All subjects were treated with 1–2 treatment courses of oral antibiotics before FMT, 8 patients (80.0%) were administered oral vancomycin, and 8 were administered metronidazole. The main manifestations of CDI symptoms were abdominal pain and diarrhea in FMT patients. Nine patients (90.0%) had prescriptions for dementia based on cognitive test results or brain imaging. The control group was not different compare to FMT group. They also included 8 women and 2 men; the median age at the time of CDI diagnosis was 77 years (range, 62–91 years). All control subjects were also treated with 1–2 treatment courses of oral antibiotics and had prescriptions for dementia.

### Clinical responses with or without FMT

A total of 10 FMT were performed; 9 (90.0%) patients were cured by a single FMT, and 1 (10.0%) was cured with two FMTs. There were no serious adverse events reported after FMT. Transient nausea was reported in 1 patient on the day of FMT and disappeared after 3 hours. Transient abdominal pain was reported in 1 patient after the procedure. In addition, 1 patient vomited during the procedure. No recurrence of CDI and FMT-related adverse events were reported by the caregivers of subjects after FMT by phone call due to COVID-19.

GCS scores were not significantly different between pre- and post-FMT. Most patients’ scores were 13 or 14 without any acute need for change in the management or neuroimaging. All patients performed significantly better on the cognitive function post-FMT than pre-FMT. Median MMSE scores were higher post-FMT (16.0, Median; 13.0–18.0, interquartile range [IQR]) than pre-FMT (10.0, Median; 7.0–14.0, IQR, Table 2 and Supplementary Table 1, Figure 1). The difference in the MMSE score pre- and post-FMT was 4.7 (mean, *P* = 0.010). As the result of Wilcoxon signed rank test pre- and post-FMT, the difference in the MMSE scores was significant (*P* = 0.005) for an average of 10 patients. Median CDR-SB score was lower post-FMT (5.5, Median; 4.0–8.0, IQR) than pre-FMT (10.0, Median 5.0–12.0, IQR, Table 2, Figure 1). The difference in the CDR-SB scores between pre- and post-FMT was 3.1 (mean, *P* = 0.048). Wilcoxon signed rank test pre- and post-FMT showed that the difference in the CDR-SB scores was significant (*P* = 0.005) in the 10 patients. In the control group, 90.0% of the patients were cured by the antibiotic treatment. Further, all control subjects did not show significantly altered cognitive function at admission and after CDI treatment (Figure 1). Median MMSE scores were rather low after CDI treatment (10.0, Median; 9.8–15.3, interquartile range [IQR]) than at admission (14.0, Median; 12.8–16.0, IQR, Table 2 and Supplementary Table 2). Median CDR-SB score also did not significantly change after CDI treatment (8.0, Median) compared to that at admission (10.00, Median, *P* = 0.470, Table 2). Thus, FMT caused a significant improvement in cognitive functions.

### Alternation of patients’ fecal composition

Twenty fecal samples were collected from 10 patients with CDI to analyze gut microbiota before and after FMT. We also collected samples from the control group at admission due to CDI and after CDI treatment. For patients treated with FMT twice, pre-FMT fecal samples were collected before the first FMT, and post-FMT samples were collected after the last FMT procedure.

**Figure 2:**
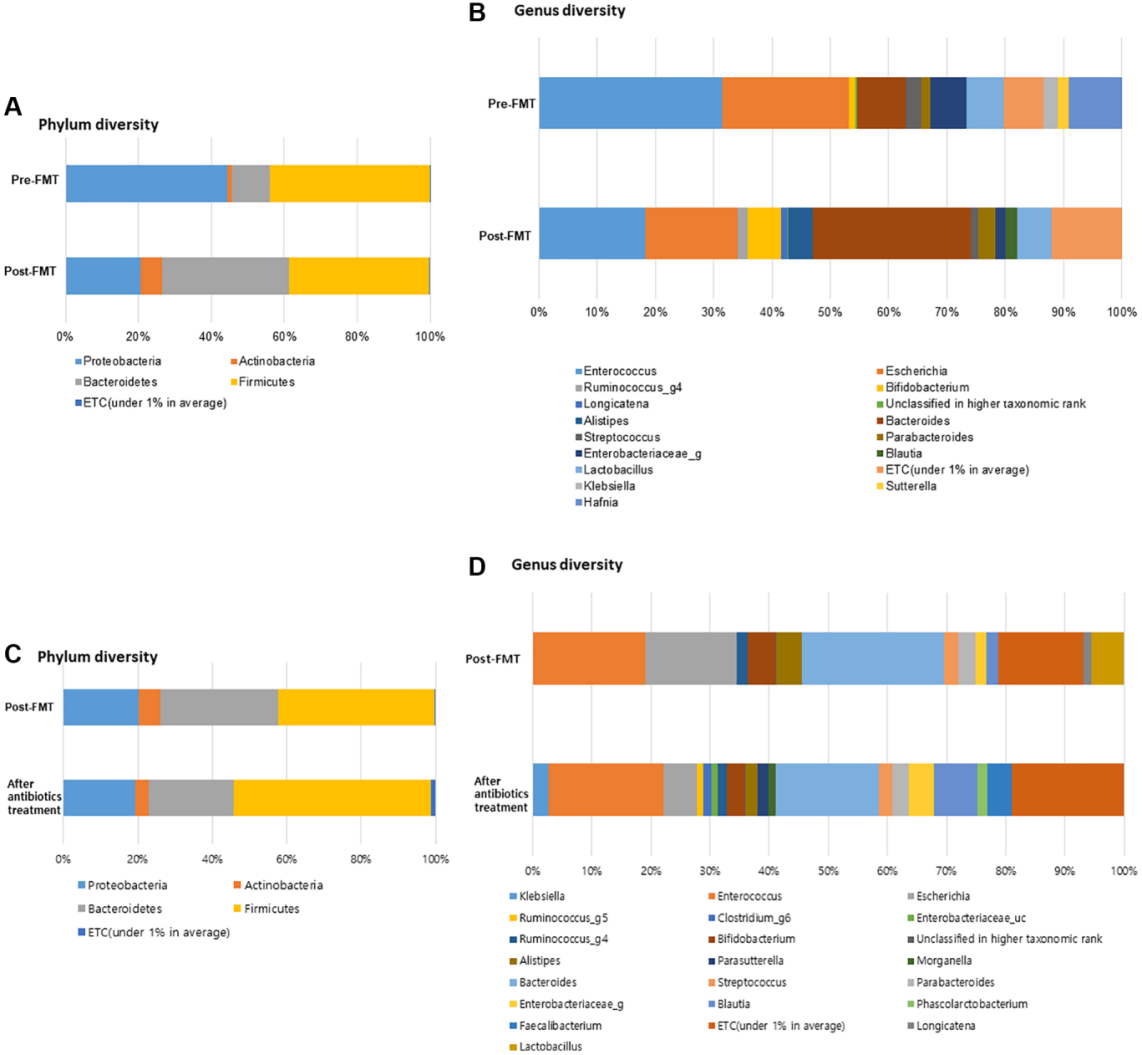
**FMT alter the gut microbiome composition in dementia patients with severe *Clostridioides difficile* infection (CDI).** (**A**) Phylum diversity pre- and post-FMT. (**B**) Genus diversity pre- and post-FMT. (**C**) Phylum diversity post-FMT and after antibiotics treatment. (**D**) Genus diversity post-FMT and after antibiotics treatment. ^*^P > 0.05, ^**^P < 0.05.

Post-FMT, the bacterial richness of gut microbiota was increased in the patients (Figure 2A, 2B). At the phylum level, *Proteobacteria* was enriched (44.25%, pre-FMT vs. 20.68%, post-FMT); moreover, *Bacteroidetes* were enriched in patients post FMT (10.44%, pre-FMT vs. 34.78%, post-FMT) (*P* = 0.893). At the genus level, relative abundances of *Hafnia* (9.08%), *Enterobacteriaceae* (6.34%), *Sutterella* (1.99%), and *Klebsiella* (2.48%) were higher in the pre-FMT samples. *Bacteroides* (8.46%, pre-FMT vs. 27.15%, post-FMT)*, Alistipes* (0.00%, pre-FMT vs. 4.18%, post-FMT)*, Blautia* (0.00%, pre-FMT vs. 2.03%, post-FMT), and *Bifidobacterium* (1.20%, pre-FMT vs. 5.78%, post-FMT) were the more enriched taxonomies after FMT (*P* = 0.161). Compared to the control group, especially after CDI treatment, in the FMT group, *Bacteroidetes* were more enriched at the phylum level (22.73% vs. 34.78%, *P* = 0.427, Figure 2C). *Firmicutes* were more enriched in the control group than in the post-FMT group (52.96% vs. 38.36%, *P* = 0.241). *Bacteroides* (17.44%, post-CDI), *Alistipes* (1.96%, post-CDI), and *Bifidobacterium* (3.02%, post-CDI) were the less enriched taxonomies at the genus level in the control group compared with post-FMT samples (*P* = 0.473, Figure 2D).

**Figure 3:**
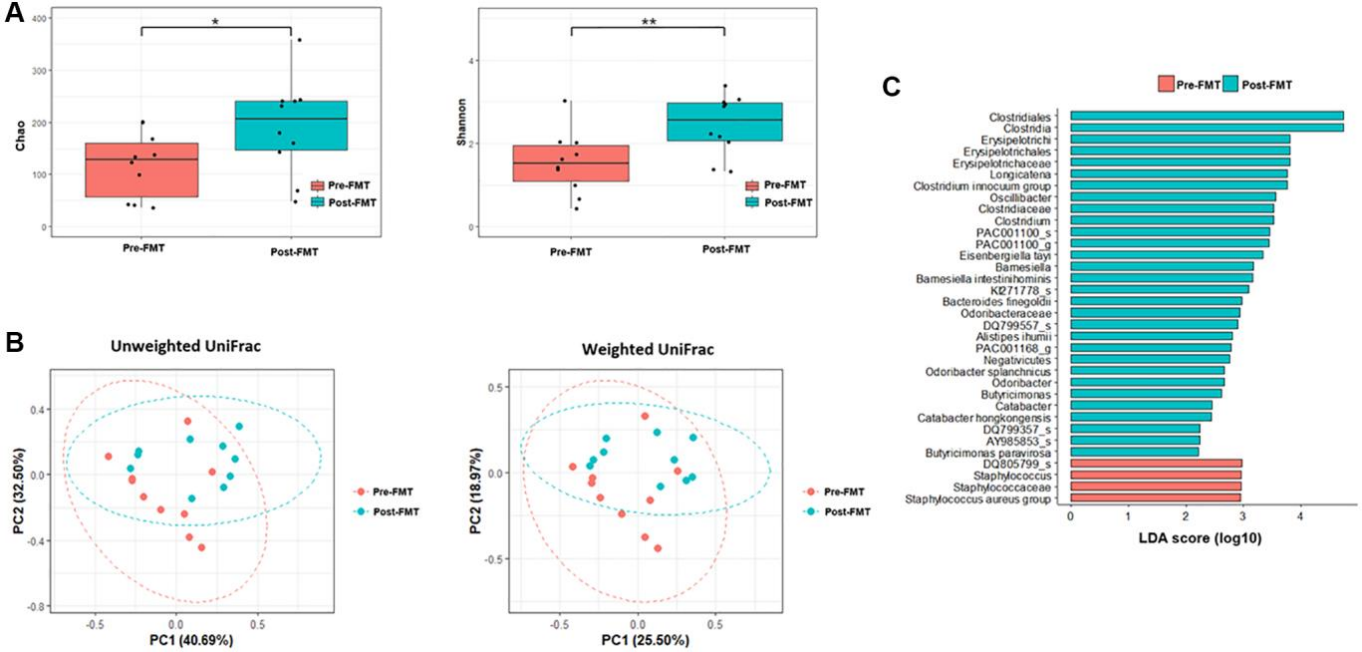
**Microbiome analysis before and after fecal microbiota transplantation (FMT).** (**A**) Alpha diversity was calculated using the number of overserved Chao and Shannon. (**B**) Principal coordinate analysis (PCoA) profile of microbial diversity across all samples using unweighted and weighted UniFrac. (**C**) Linear discriminant analysis effect size analysis (*P* < 0.05). ^*^P > 0.05, ^**^P < 0.05.

**Figure 4:**
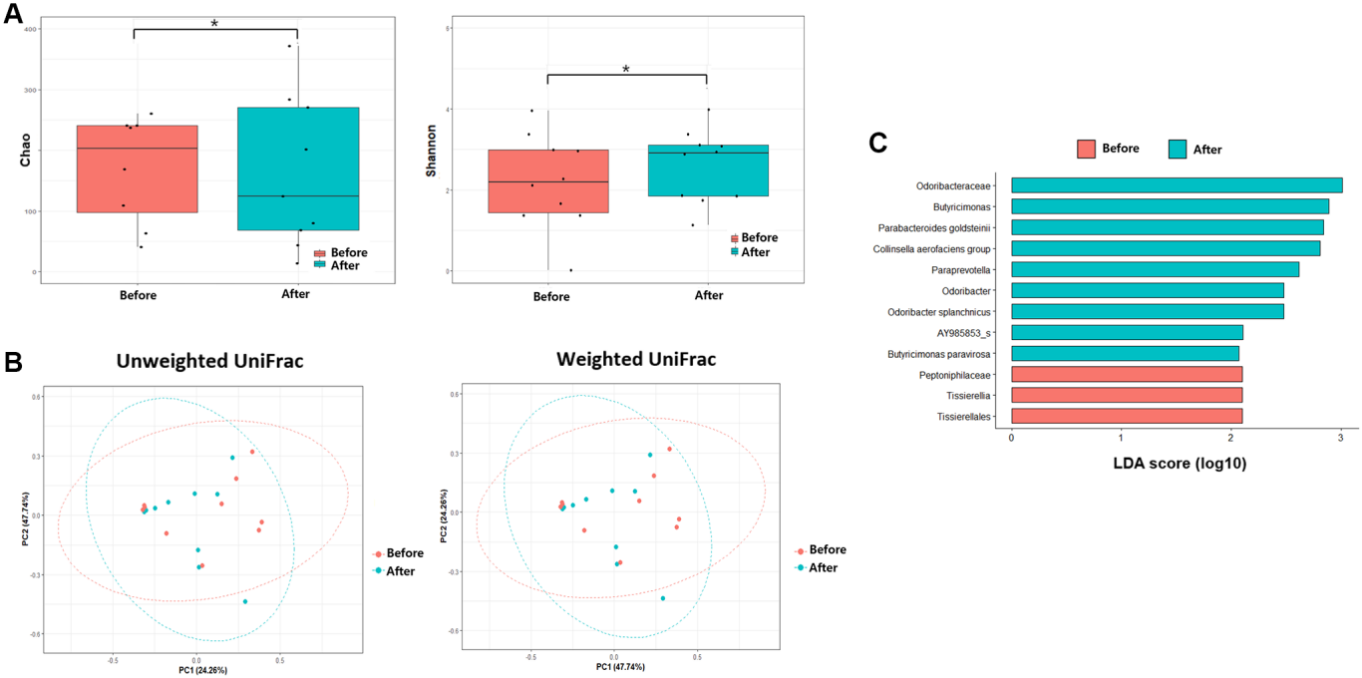
**Microbiome analysis before antibiotics treatment and after antibiotics treatment.** (**A**) Alpha diversity was calculated using the number of overserved Chao and Shannon. (**B**) Principal coordinate analysis (PCoA) profile of microbial diversity across all samples using unweighted and weighted UniFrac. (**C**) Linear discriminant analysis effect size analysis (*P* < 0.05). ^*^P > 0.05.

The alpha diversities were significantly different before and after FMT (Chao, *P* = 0.11; Shannon, *P* = 0.026, Figure 3). In the control group, the alpha diversities were not significantly different at admission and after CDI treatment (Chao, *P* = 0.650; Shannon, *P* = 0.496, Figure 4). In other words, the alpha diversity indices were increased after FMT. This indicates that FMT changes the species composition of recipient feces. The beta diversity showed a significant difference between pre- and post- FMT groups according to the permutational multivariate analysis of variance (PERMANOVA, *P* = 0.046, Figure 3) [10]. There were no significant differences in beta diversity at admission and after CDI treatment according to PERMANOVA (*P* = 0.659, Figure 4).

Inter-group comparisons of taxonomic profiles revealed that the gut microbiome altered in the abundance of several taxa. LEfSe was performed to determine the significant taxonomic difference between pre- and post-FMT (Figure 3). After FMT, some bacteria were uplifted or reduced. At the phylum level, *Bacteroidetes* was the more enriched taxonomy after FMT (LEfSe >2, *P* < 0.05). At the genus level, *Longicatena, Barmesiella,* and *Odoribacter* were also enriched taxonomy after FMT (LEfSe >2, *P* < 0.05). In the control group, *Bacteroidota* and *Actinomycetota* were the more enriched taxonomies at the phylum level after CDI treatment (LEfSe >2, *P* < 0.05, Figure 4). At the genus level, *Butyricimonas* and *Odoribacter* were enriched after CDI treatment (LEfSe >2, *P* < 0.05, Figure 4).

**Figure 5:**
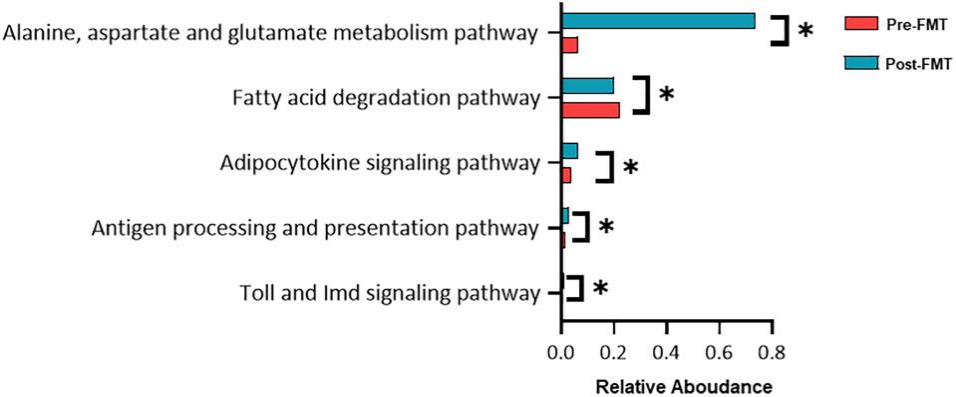
**Functional composition difference pre- and post-fecal microbiota transplantation (FMT).** Relative abundances of the most abundant microbial pathways among the different groups. Data shown are the means ± SD. ^*^P < 0.05.

Functional biomarker analysis using the Kruskal-Wallis H test was performed between the pre- and post-FMT groups. Alanine, aspartate, and glutamate metabolism pathways were found to be significantly modulated between before and after FMT (Figure 5, *P* = 0.034).

## DISCUSSION

To our best knowledge, this is an interesting study to describe cognitive improvements in dementia patients with recurrent severe CDI after FMT. In our study, the MMSE, CDR-GS, and CDR-SB scores improved in all patients after FMT. Alterations in gut microbiome composition consistently influenced the gut microbiome, which may be closely related to cognitive function. Therefore, this study provides clinical evidence in support of the brain-gut-microbiome association. Improving cognitive function by modulating the gut microbiota is a novel area for the bidirectional communication pathway, and this study is a meaningful presentation of a link between the brain and gut [11].

Dementia is a neurodegenerative disorder, specifically accompanied by progressive cognitive decline. Effective and definitive therapeutic management for cognitive decline has not yet been established. Therefore, various mice model was used to investigate dementia treatments [12]. Previous studies have demonstrated the relationship between cognitive function and gut microbiome in animal models [6]. Since most animal models are based on gene modification and validation, the limitation generated to demonstrate disease’ onset and similarity of humans. However, we constructed dementia patients with FMT to correlate if pathogenesis affected cognition.

The study results suggest that FMT positively affects cognitive function. Gut microbial diversity was increased after FMT. Indeed, the gut microbiota of dementia patients had low diversity compared to healthy people, which is consistent with the findings of previous animal model studies [5, 13]. In addition, a dramatic increase in *Proteobacteria* and *Bacteroidetes* was observed before and after FMT, respectively. Primarily, these genera are associated with positive effects in cognitive function [14–17]. In addition, *Longicatena, Barmesiella,* and *Odoribacter* are associated with cognitive function at the genus level. These microbiomes improve the cognitive function in AD individuals. On the contrary, *Butyricimonas* and *Odoribacter* are correlated with cognitive dysfunction [18], suggesting that the microbiome composition changes after FMT as compared to CDI treatment without FMT. These results suggest that altered fecal microbiome composition affects cognitive function. The intervention of microbiome composition, such as FMT, could affect cognitive function via many metabolic pathways. Therefore, the gut microbiome might influence the pathophysiological effects of cognitive function through various mechanisms.

Our study hypothesized that gut microbial composition affects the gut metabolite pathway and alters the gut microbiome affecting the brain, such as bile acids, amino acids, and short-chain fatty acids. Alternation of gut metabolite also might correlate to changes in the gut microbiota. Among various gut metabolite, changes in amino acid metabolism have been observed in AD brain [19, 20]. Recent metabolomics studies showed changes in the levels of various amino acids in the brain and plasma of AD patients. It is essential to consider that changes in amino acid metabolism are a driving force for disease progression. Therefore, the balance of amino acid metabolites is associated with dementia progression [21–26]. Further investigation of the role for metabolic dysregulation of amino acids in AD pathogenesis is needed [27–31].

This study showed that alanine, aspartate, and glutamate (amino acid metabolite) levels differed after FMT. These amino acids have been shown to decrease in the brain and plasma of patients with AD, particularly in the temporal lobe cortex [22–34]. Reduced glutamate levels are associated with impaired cognitive function, while decreased hippocampal glutamate levels are associated with episodic memory performance in dementia, including mild cognitive impairment. During gut metabolism, microbiome-mediated molecular mimicry may be responsible for decreased levels of amino acids essential for the normal brain function [35]. Because molecular mimicry occurs between brain and gut microbiota in cross-reaction immune cells including T or B cells, gut microbiota is considered a well-assumed strategy for increasing gut microbiota metabolites.

Importantly, these results imply that changing the amino acid composition affects glucose metabolism and eventually, the brain. AD is associated with dysfunctional energy metabolism and neurons contain the fatty acid beta-oxidation enzymes. Amino acid catabolism, including lactate and glucose metabolism, plays an important role in maintaining cellular adenosine triphosphate (ATP) levels in AD neurons [36, 37]. Therefore, FMT could change the amino acid metabolite composition and affect the brain. This study showed that FMT affects amino acid metabolism, especially affecting cognition [19, 20].

Another possible clue is that amyloid plaques in the brain might be metabolized by the gut microbiota [34]. Amyloid plaques, including amyloid-beta (Aβ), are pathophysiologically associated with neuroinflammation. Previous studies have reported microbe- associated and dementia-associated Aβ signals, required for the microglia activation, through the same Toll-like receptor (TLR)2/TLR1 complex [37]. Aβ deposition affects microglial activation; therefore, it has emerged as a key factor in AD pathogenesis. Aβ accumulation and microglia activation are present in the brain of AD patients. Aβ proportion was also found to be increased in a mouse model with cognitive impairment, as observed in humans [38, 39]. FMT improved the cognitive deficits and reduced Aβ deposition in mice [6]. Injection of healthy mouse feces into the gut of cognitively impaired mice resulted in an improvement in the cognitive functions. Further, a reduction in Aβ accumulation was also observed. FMT can downregulate pro-inflammatory cytokines and inhibit protein transcription factors elevated in AD [2, 40]. This gut microbiota modulate recipient homeostasis via the blood-brain barrier and intestinal integrity after FMT, resulting in improved cognitive function.

In our study, the attention and calculation domains and daily living abilities of patients significantly improved after FMT. A previous study demonstrated that microbial dysbiosis increased hippocampal neurodegeneration and disrupted neurogenesis [41]. Thus, alterations in microbiome composition have been associated with cognitive activity, such as learning, environmental enrichment, and exercise. Further evaluation is necessary to correlate unique regions of cognitive domains and FMT. This report also presents a patient (Case 3) who experienced a step-wise improvement in the cognitive function following repeated FMT in cognitive domains of orientation, attention, and short-term memory, in addition to daily living ability [14]. Whether FMT has a continuous and cumulative effect on cognitive function is challenging to confirm.

To determine whether the cognitive function is affected by patients' general condition, laboratory findings were also checked 1 week before and after FMT. The laboratory findings suggested that patients’ medical condition did not significantly affect cognitive function before and after FMT (Table 3). Therefore, this study provided clinical evidence indicating improved cognitive function after FMT and comparatively improved cognitive function within a short time after FMT administration.

**Table 3:** Laboratory findings in the FMT group.

**Value (before FMT/after FMT)**	**Case**										
	**1**	**2**	**3-1**	**3-2**	**4**	**5**	**6**	**7**	**8**	**9**	**10**
**BUN, mg/dL** **(normal range: 6.0–20.0)**	13.3/3.7	7.7/6.7	37.2/26.8	23.4/16.6	2.1/1.9	23.4/12.8	11.8/12.8	18.6/22.7	23.6/12.0	10.7/13.1	7.3/6.5
**Creatinine, mg/dl** **(normal range: 0.5–1.20)**	0.36/0.30	0.57/0.39	1.88/1.97	2.27/2.09	0.45/0.32	1.07/0.79	0.75/0.79	0.57/0.58	2.13/2.07	0.77/0.85	0.26/0.28
**CRP, mg/dl** **(normal range: 0.0–0.5)**	6.34/4.51	1.39/0.13	1.20/3.60	6.17/2.29	1.56/2.28	0.63/1.43	0.75/0.54	3.25/5.94	3.12/0.70	9.29/5.87	1.06/1.78
**ESR, mm/hr** **(normal range: 1–22)**	22.0/7.0	37.2/26.8	37.2/26.8	67/NA	21.0/50.0	63.0/101.0	20.0/121.0	16.0/7.0	50.0/41.0	69.0/43.0	14.0/8.0
**Procalcitonin, ng/ml** **(normal range: 0.0–0.50)**	NA/0.07	0.37/0.09	0.73/0.14	0.10/0.14	0.71/0.47	0.79/0.14	0.06/0.06	0.68/4.79	0.14/0.19	0.18/0.15	0.08/0.07
**Ammonia, μg/dl** **(normal range: 12–66)**	35.0/NA	67.0/NA	42.0/NA	NA/NA	NA/19.0	NA/NA	NA/NA	NA/NA	NA/NA	39.0/NA	NA/NA
**Albumin, g/dL** **(normal range: 3.5–5.2)**	1.9/1.8	3.5/2.8	2.7/2.7	2.7/3.4	2.1/2.3	3.0/3.1	3.0/2.6	1.9/1.9	2.7/2.5	2.9/2.7	3.0/3.1
**Lactic acid, mmol/L** **(normal range: 0.5–2.20)**	1.40/NA	NA/1.52	2.22/1.21	0.76/1.38	1.13/1.02	1.44/1.23	0.84/1.23	2.37/1.29	1.19/0.73	1.38/0.99	0.92/NA

Abbreviations: FMT: fecal microbiota transplantation; NA: not applicable.

This study has limitations. First, we adjusted the follow-up timing for cognitive function analysis to reduce the bias that appears to improve cognitive function. Therefore, we checked the patients’ laboratory results before and after FMT. Second, this study could not perform various cognitive function tests due to hospital policy regarding CDI patients. Patients with CDI have limited permission to go outside the hospital room except for important evaluations. Therefore, we did limited cognitive tests. Although MMSE was limited, it was clinically useful [40]. In addition, MMSE could evaluate the mild-to-moderate stages of dementia [41]. Enrolled patients in our study had mild-to-moderate stages of dementia, and hence, MMSE was implemented. In addition, to reinforce MMSE’s accuracy, we additionally used CDR because it is associated with good interrater reliability and criterion validity [42]. However, a more diverse set of cognitive function tests must be evaluated in future studies to confirm our findings. Third, it is necessary to analyze gut microbiome differences, taxa, and additional mechanisms in patients with cognitive decline and cognitive improvement for larger patient groups and multiple centers. In addition, the FMT results are not consistent across studies, likely due to the different methodologies and instrumentation used. Since we did not check if enteric neuron receptors of FMT promoted metabolite composition and affected gastrointestinal motility, further studies are needed on transmitting information of metabolites across the epithelium to the brain barrier and changing concentrations of metabolites in the intestinal lamina propria. Fourth, although FMT is a relatively safe procedure and our patients did not present with fatal side effects, adverse effects such as fever, abdominal pain, and abdominal distension can occur after FMT (Table 1). Therefore, patient selection before FMT and monitoring after FMT is necessary. Fifth, although we analyzed laboratory tests before and after FMT, various general conditions (diarrhea, fever, nutrition status, ambulation, mood, etc.) and medical conditions can affect cognitive function. Most patients were on oral feeding (90%) in this study. However, there are individual differences in the amount eaten, which could not reflect the required daily energy that was enough for individual patients. Therefore, there seems to be a need for a further study to consider the association between patient’s nutrition support status and gut microbiome before and after FMT. In addition, because of the COVID-19 pandemic and associated hospital policies, we could perform limited test for patients. Therefore, we need to further evaluate the patient’s mood and functional activity changes. Finally, we did not evaluate dementia using other diagnostic tools such as the brain magnetic resonance imaging (MRI). Because we assessed the patients’ cognition before and after 1 month of FMT, the duration was short to perform brain MRI during follow-up. In addition, it was difficult to show the change in GCS scores before and after FMT because it demonstrates a state of consciousness and is mainly used in trauma patients [42]. Therefore, we plan to evaluate the cognition change after FMT during long-term follow-up in future studies.

FMT might affect the dysbiosis of the patient’s microbiome. We suggest that FMT alters the diversity and specificity of gut microbiota, which affects the brain-gut-microbiome axis. The positive effects of FMT may be associated with changes in the gut microbiota and an increase in profitable brain metabolites, similar to those found in mice studies [43]. This study suggests that FMT is a viable treatment option for patients with cognitive decline, thereby providing a new opportunity for practical improvements in patients with dementia. It also further highlights the critical importance of the association between the gut microbiome and cognitive function.

## MATERIALS AND METHODS

### Patients

Patients were screened and enrolled between November 2019 and July 2021 via the Dementia and Microbiome centers of Inha University Hospital, Incheon, Republic of Korea. We initially screened patients admitted to our institution because of dementia diagnosis by a neurologist, especially Alzheimer’s dementia (AD). Simultaneously, we screened for severe CDI patients who had previously failed to improve with several antibiotics and had a relapse of symptoms with a positive stool test [44]. Patients were enrolled if they met all the following inclusion criteria: 1) over 19 years of age, 2) with dementia, 3) with CDI, and 4) absolutely required FMT. Patients who were unsuitable for FMT due to severe systemic disease (immunocompromised, acute inflammation, or infectious diseases) and those whose cognitive function could not be tested were excluded initially. Sixty-eight patients with written informed consent were enrolled; however, 27 were lost to follow-up, and 31 were not appreciate fecal analysis due to patients’ fecal samples (Supplementary Table 3). Consequently, 10 patients were enrolled for the analysis. Additionally, we matched 10 dementia patients with CDI having similar characteristics as that of the FMT group. They were treated with antibiotics and enrolled as the control group using 90% power and 5% Type I error rate.

### Fecal microbiota transplantation

Stool donors with no gastrointestinal problems or other health problems underwent blood and stool tests and responded to a specific questionnaire for FMT donor selection [44]. Informed consent was obtained before screening procedures. Donated stool (50–70 g) was filtered to make a stool suspension and stored at −80°C. Thereafter, the stool suspension was strained and poured into a sterile container. Stool suspensions (60 g/300 mL) were thawed at 37°C for 6 h before FMT. Recipients’ antibiotic prescriptions for CDI were stopped 48 h before the FMT procedure. Recipients underwent bowel preparation according to doctor’s instructions before FMT. Stool suspensions (60 g) were injected into the patient through a colonoscopy by a skilled gastroenterologist, following evidence-based FMT guidelines [44]. After FMT, recipients were monitored for any side effects of FMT at the microbiome center.

### Cognitive function and laboratory evaluations

Cognitive function and mental status evaluations were performed individually on all dementia patients for approximately 20–30 min by the same neurologist. The MMSE (range, 0–30), CDR (range, 0–3), and GCS (range, 3–15) were administered 1 month before and 1 month after FMT in the FMT group. Control group patients were also assessed at admission due to CDI and 1 month after CDI. The sensitivity and specificity of the MMSE are 27% to 89% and 32% to 90%, respectively [45]. Therefore, MMSE had limited diagnostic accuracy, especially for distinguishing mild cognitive impairment from AD dementia [45]. Although MMSE has limitations, we used it because it has long been used to detect and monitor dementia progression. The CDR has been widely used and has been validated as a reliable tool to grade dementia severity [46, 47]. The CDR scale yields Sum of Boxes (CDR-SB) scores (range, 0–18) with the global score regularly used in clinical and research settings to stage dementia severity [17, 45]. Higher scores on the MMSE and lower scores on CDR-SB suggest better cognitive performance. The GCS is the sum of the scores as well as the individual elements (3 being the worst and 15 being the highest) [41]. For example, a score of 12 might be expressed as GCS12 = E4V5M3. We performed laboratory tests before and after FMT or at admission due to CDI and after CDI treatment at the same time as possible when performing cognitive function tests (maximum 3 days before and after cognitive test) to further confirm whether the patients' cognition is affected by the general condition.

### Microbiome analysis

We collected patients’ fecal samples before FMT and compared them with the microbiota compositions 3 weeks later. Fecal samples of control group patients were also collected at admission and 3 weeks after the CDI treatment. All samples were stored at −80°C until shipping to the Macrogen Biotech Lab (Seoul, Korea) for DNA extraction and sequencing. Metagenomic DNA was extracted from fecal samples, and amplification of the V3-V4 region of the bacterial 16S rRNA gene was conducted using barcoded universal primers. Sequencing was carried out using a MiSeq sequencer on the Illumina platform (Macrogen Inc., Seoul, Korea) according to the manufacturer’s specifications. Microbiome profiling was conducted with the 16S-based Microbial taxonomic profiling (MTP) platform of EzBio-Cloud Apps (ChunLab Inc., Seoul, Korea). After taxonomic profiling of each sample, the comparative MTP analyzer of EzBioCloud Apps was used for comparative analysis of the samples. In the MTP platform of ChunLab Inc., Seoul, Korea, preprocessing of the sequencing reads was conducted using the following five steps: 1) filtering of low-quality reads, 2) merging of the paired-end reads, 3) removal of barcode and primer sequences, 4) taxonomic assignment of the reads, and 5) removal of chimeric sequences. Taxonomic assignment of the reads was conducted with ChunLab’s 16S rRNA database (DB ver. PKSSU4.0) [48]. OUT picking was conducted with UCLUST and CD-HIT with a 97% similarity cutoff [49]. Then, Good’s coverage, rarefaction, and alpha-diversity indices, including Chao and Shannon found in MTP, were calculated. Beta-diversity was analyzed using the PERMANOVA test [10]. Overall differences in the microbiome structure were evaluated through Principal Coordinate Analysis (PCoA) to unweighted and weighted UniFrac distance [50, 51]. Linear discriminant analysis (LDA) effect size (LEfSe) was performed to determine the taxonomic difference between pre- and post-FMT [52]. Functional biomarker analysis using phylogenetic investigation of communities by reconstruction of unobserved states (PICRUSt) software package was performed between the pre and post-FMT groups using the EzBio-Cloud Apps [53].

### Outcome

The primary end point was a correlation between FMT and cognitive function in dementia patients with CDI compared to the control group. In addition, we analyzed the patients’ gut microbiome before and after FMT to investigate microbiome metabolites that affect cognition.

### Statistical analysis

Data are presented as the mean, range, median, or number (percentage) as appropriate. Statistical significance between the two groups was determined using the Kruskal-Wallis test, a non-parametric method for testing whether samples originate from the same distribution. In addition, between two groups comparison were conducted using the Wilcoxon signed rank test, a non-parametric statistical test. Statistical analysis was performed using GraphPad Prism (Version 9.0, GraphPad Software, San Diego, CA, USA), R statistics package (R Foundation, Vienna, Austria; https://www.r-project.org), and SPSS 26.0 for Windows (SPSS Inc., Chicago, IL, USA). *P* values < 0.05 were considered significant.

## Supplementary Materials

Supplementary Tables
