# Supplementary material for: Fecal microbiota transplantation can improve cognition in patients with cognitive decline and Clostridioides difficile infection
Source: Aging (Albany NY). 2022 Aug 16;14(16):6449–66. doi: 10.18632/aging.204230 (PMC9467396; doi:10.18632/aging.204230)
Supplement: Supplementary Tables [file aging-14-204230-s001.pdf]

SUPPLEMENTARY TABLES

Supplementary Table 1. Cognitive function difference between before and after fecal microbiota transplantation.

Characteristics	Case										
	**1**	**2**	**3–1**	**3–2**	**4**	**5**	**6**	**7**	**8**	**9**	**10**
**GCS (before FMT/after FMT)**	14/14	13/14	14/14	14/14	14/14	13/14	13/14	13/14	14/14	13/14	13/13
**MMSE (before FMT/after FMT)**											
Total	8/13	13/17	15/18	20/20	14/19	5/12	7/14	10/16	13/17	10/14	7/9
Orientation to time (5)	2/2	2/2	3/3	3/3	3/3	2/3	1/2	1/1	2/3	0/2	1/2
Orientation to place (5)	2/2	3/2	2/2	3/3	2/2	1/2	2/4	4/5	4/4	3/3	2/3
Registration (3)	1/2	1/2	3/3	3/3	2/3	0/2	2/3	3/3	3/3	3/3	1/1
Attention and calculation (5)	1/4	2/5	2/4	4/4	2/4	1/1	0/0	0/1	1/1	1/2	1/1
Recall (3)	1/1	1/2	1/2	3/3	2/3	0/2	1/1	0/1	1/2	0/1	0/1
Language (8)	1/1	3/3	3/3	3/3	3/3	1/3	1/4	2/5	2/4	3/3	2/1
Visual Construction (pentagon) (1)	0/1	1/1	1/1	1/1	0/1	0/0	0/0	1/0	0/0	0/0	0/0
**CDR-GS (before FMT/after FMT)**											
	2/1	1/1	1/1	0.5/0.5	1/0.5	2/1	2/1	2/1	1/0.5	2/1	2/2
Memory	2/1	1/1	1/1	0.5/0.5	0.5/0.5	2/1	2/1	3/1	1/0.5	1/0.5	2/2
Orientation	2/2	1/1	1/1	0.5/0.5	1/0.5	2/2	2/2	2/1	1/0.5	1/0.5	3/2
Judgment problem solving	1/1	1/1	1/0.5	1/1	1/1	2/1	2/1	2/1	1/1	2/0.5	3/3
Community affairs	2/2	1/1	1/1	1/1	1/1	2/2	2/2	2/1	1/1	2/1	2/1
Home and hobbies	2/1	2/1	0.5/0.5	0.5/0.5	0.5/0.5	2/1	2/1	2/0.5	1/0.5	3/1	2/1
Personal care	1/1	1/1	1/1	0.5/0.5	1/0.5	1/1	2/1	2/1	1/0.5	3/1	2/2
**CDR-SB score (before FMT/after FMT)**											
	10/8	7/6	5.5/5	4/4	5/4	11/8	12/8	13/5.5	5/4	12/4.5	14/11

Abbreviations: GCS: Glasgow Coma Scale; FMT: fecal microbiota transplantation; MMSE: Mini-mental state examination; CDR-GS: Clinical dementia rating global score; CDR-SB score: Clinical Dementia Rating Scale Sum of Boxes Score.

Supplementary Table 2. Cognitive function difference in dementia patients with severe CDI without fecal microbiota transplantation.

Characteristics	Case									
	**1**	**2**	**3**	**4**	**5**	**6**	**7**	**8**	**9**	**10**
**GCS (before antibiotics treatment/ after antibiotics treatment treatment)**	14/14	13/14	14/14	14/14	13/14	13/14	13/14	14/14	13/14	13/13
**MMSE (before antibiotics treatment/after antibiotics treatment)**										
Total	14/15	14/10	10/10	16/15	15/16	12/9	16/17	16/10	13/7	14/10
Orientation to time (5)	3/3	2/2	3/3	3/3	3/3	2/2	4/4	4/2	3/2	4/2
Orientation to place (5)	3/3	3/2	2/2	3/2	3/3	3/2	4/3	4/2	3/2	3/3
Registration (3)	1/2	2/2	1/1	2/2	3/3	2/1	3/3	3/3	3/0	2/1
Attention and calculation (5)	1/1	2/1	2/1	2/2	2/1	1/0	1/1	1/1	1/1	1/1
Recall (3)	2/2	1/2	1/1	2/2	1/2	1/0	1/1	1/1	0/0	1/1
Language (8)	4/4	3/1	1/2	4/4	3/3	3/4	2/4	2/1	3/2	3/2
Visual Construction (pentagon) (1)	0/0	1/0	0/0	0/0	0/0	0/0	1/1	1/0	0/0	0/0
**CDR-GS (before antibiotics treatment/after antibiotics treatment)**										
	1/1	2/1	2/2	1/1	1/1	2/2	1/1	1/1	2/1	1/1
Memory	2/1	2/1	2/2	1/1	2/1	2/2	1/1	1/2	1/1	1/2
Orientation	2/2	1/1	2/2	1/1	1/1	2/2	2/2	1/2	1/2	2/1
Judgment problem solving	1/1	2/2	2/2	1/1	2/1	2/3	1/1	2/1	2/2	2/2
Community affairs	1/1	2/1	2/2	1/1	1/2	2/2	2/1	1/1	2/1	2/1
Home and hobbies	2/2	1/1	3/3	0.5/0.5	2/2	2/2	2/2	0.5/0.5	3/3	1/1
Personal care	1/1	2/2	3/3	1/1	1/1	2/3	2/1	1/1	2/3	2/2
**CDR-SB score (before antibiotics treatment/after antibiotics treatment)**										
	9/8	10/8	14/14	5.5/5.5	9/8	12/14	10/8	6.5/7.5	11/12	10/9

Abbreviations: CDI: *Clostridioides difficile* infection; GCS: Glasgow Coma Scale; FMT: fecal microbiota transplantation; MMSE: Mini-mental state examination; CDR-GS: Clinical dementia rating global score; CDR-SB score: Clinical Dementia Rating Scale Sum of Boxes Score.

Supplementary Table 3. Clinical characteristics of dropout patients.

Characteristics	Value (*n* = 58)
**Sex, female, *n* (%)**	32 (55.2)
**Age, years, median (IQR)**	74 (63–90)
**mRS, median (IQR)**	3 (1–4)
**MMSE, median (IQR)**	13.0 (4.9–15.8)
**CDR-SB score, median (IQR)**	10.0 (8.2–11.1)
**Nutrition supplement status**	
Oral feeding, *n* (%)	42 (72.4)
Enteral feeding, *n* (%)	13 (22.4)
Peripheral feeding, *n* (%)	3 (5.2)
**Presenting symptoms of CDI**	
Diarrhea, *n* (%)	42 (72.4)
Abdominal pain, *n* (%)	38 (65.5)
Fever, *n* (%)	12 (20.7)
**Antibiotics for CDI**	
Vancomycin, *n* (%)	34 (58.6)
Metronidazole, *n* (%)	16 (27.6)
Vancomycin + metronidazole, *n* (%)	8 (13.8)
**Medication for dementia**	
Donepezil, *n* (%)	32 (55.2)
Memantine, *n* (%)	8 (13.8)
Donepezil + Memantine, *n* (%)	18 (31.0)
**Duration of dementia, years, median (IQR)**	4.5 (1.9–6.7)
